# Biological evaluation of a glucose‐based boron carrier as a potential agent for boron neutron capture therapy

**DOI:** 10.1002/ijc.70054

**Published:** 2025-07-23

**Authors:** Surachet Imlimthan, Katayun Bahrami, Henna Pehkonen, Alessia Centanni, Ahmed B. Montaser, Arina Värä, Jelena Matović, Heidi Liljenbäck, Tatsiana Auchynnikava, Kristiina M. Huttunen, Anne Roivainen, Anu J. Airaksinen, Filip S. Ekholm, Outi Monni, Jarkko Rautio, Mirkka Sarparanta

**Affiliations:** ^1^ Department of Chemistry University of Helsinki Helsinki Finland; ^2^ School of Pharmacy University of Eastern Finland Kuopio Finland; ^3^ Applied Tumor Genomics Research Program, Faculty of Medicine University of Helsinki Helsinki Finland; ^4^ Turku PET Centre and Turku Center for Disease Modeling University of Turku Turku Finland; ^5^ Turku PET Centre and Department of Chemistry University of Turku Turku Finland; ^6^ Applied Tumor Genomics Research Program and Department of Oncology, Clinicum, Faculty of Medicine University of Helsinki Helsinki Finland

**Keywords:** BNCT, boron delivery agents, boron neutron capture therapy, drug delivery, glucoconjugate, glucose transporter 1, GLUT1

## Abstract

Boron neutron capture therapy (BNCT) is an innovative radiation oncology approach that targets tumors selectively, minimizing damage to healthy tissues through high‐linear‐energy‐transfer particles released during the boron neutron capture reaction. Current boron carriers like sodium mercaptoundecahydrododecaborate (BSH) and L‐*p*‐boronophenylalanine (BPA) face limitations in specificity and solubility. Our recently developed 6‐*O*‐(*o*‐carboranylmethyl)‐d‐glucopyranose (B‐Glc) shows promise as an alternative, demonstrating strong interactions with glucose transporters in human head and neck squamous cell carcinoma (HNSCC) CAL 27 cells in vitro. This study aims to extend in vitro investigations to three additional patient‐derived human HNSCC cell lines (UT‐SCC‐14, UT‐SCC‐28, and UT‐SCC‐42B) and to further evaluate in vivo pharmacokinetics in selected HNSCC tumor xenografts. The B‐Glc showed superior uptake and favorable kinetic parameters compared to BPA and BSH in all tested cell lines. Initial positron emission tomography imaging using [^18^F]fluoro‐2‐deoxy‐d‐glucose ([^18^F]FDG) radiotracer confirmed increased glucose uptake in CAL 27 and UT‐SCC‐14 tumors in vivo, supported by glucose transporter 1 (GLUT1) expression observed in tumor section immunohistochemistry. Biodistribution studies of the B‐Glc (75 mg/kg dose) revealed no significant impact of blood glucose levels on tumor uptake, with peak boron accumulation at 15–30 min post‐injection, comparable uptake to the clinical BPA‐fructose complex (400 mg/kg dose) performance at 60 min, achieving the required tumor boron concentration (>20 ppm) for effective BNCT. Overall, this study underscores an advancement in targeted BNCT, highlighting B‐Glc as an effective GLUT1‐targeting carrier for enhanced therapeutic outcome in HNSCC and the potential to use [^18^F]FDG as a companion diagnostic for the glucoconjugate.

## What's New?

Boron neutron capture therapy (BNCT) is a targeted form of radiation therapy designed to selectively destroy cancer cells. BNCT is not widely used, however, owing in part to challenges involving selective boron delivery to tumors. The present study evaluated the potential of a novel glucoconjugate, 6‐*O*‐(*o*‐carboranylmethyl)‐d‐glucopyranose (B‐Glc), as an alternative boron carrier for BNCT. B‐Glc demonstrated effective uptake in vitro and in vivo, marked by strong interaction with its targeted transporter, GLUT1. In vivo B‐Glc performed comparably to existing boron‐delivery agents but at a lower dose. The findings highlight the promise of B‐Glc as a viable boron‐delivery agent in BNCT.

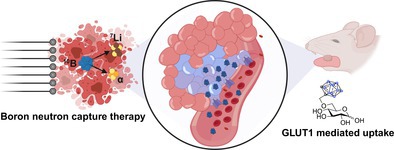

**Abbreviations**
α: Alpha particle
[^18^F]FDG: 2‐[^18^F]fluoro‐2‐deoxy‐d‐glucose
%ID/g: Percent injected dose per gram
^10^B: Boron‐10
^4^He: Helium‐4
^7^Li: Lithium‐7
ATP: Adenosine triphosphate
B‐Glc: 6‐*O*‐(*o*‐carboranylmethyl)‐d‐glucopyranose
BNCT: Boron neutron capture therapy
BPA: L‐*p*‐boronophenylalanine
BPA‐F: L‐*p*‐boronophenylalanine‐fructose complex
BSH: Sodium mercaptoundecahydrododecaborate
CO_2_
: Carbon dioxide
CT: Computed tomography
DMSO: Dimethyl sulfoxide
DPBS: Dulbecco's phosphate‐buffered saline
GBq: Gigabecquerel
GLUT1: Glucose transporter 1
H&E: Hematoxylin and eosin
HBSS: Hank's balanced salt solution
HNSCC: Head and neck squamous cell carcinoma
IC_50_
: Half‐maximal inhibitory concentration
*K*_*m*_
: Michaelis constant
LET: Linear energy transfer
MBq: Megabecquerel
n.s.: Not significant
PET: Positron emission tomography
p.i.: Post‐injection
ppm: Parts per million
ROS: Reactive oxygen species
rpm: Rounds per minute
RT: Room temperature
SUV: Standardized uptake value
UT‐SCC: University of Turku‐squamous cell carcinoma
*V*_max_
: Maximum velocity

## 1 INTRODUCTION

Boron neutron capture therapy (BNCT) has evolved over several decades as a potent treatment in clinical radiation oncology.^1^, ^2^ However, it has not been widely adopted globally due to the restricted clinical availability of neutron irradiation facilities and the limited number of effective boron delivery agents. Compared to other therapeutic approaches, BNCT demonstrates potential by selectively damaging cancer cells where the boron containing drugs have accumulated while preserving healthy tissues, positioning it as a promising treatment option for various cancers,^3^, ^4^, ^5^, ^6^ particularly head and neck squamous carcinoma (HNSCC).^7^, ^8^ The treatment typically begins with the administration of a non‐radioactive boron (^10^B) or ^10^B cluster containing targeting agents, which should display high affinity and rapid accumulation to the tumor. Subsequently, BNCT involves thermal neutron irradiation at the tumor site, initiating a boron neutron capture reaction that produces two fission products: (1) a high linear energy transfer (LET) α particle (^4^He) and (2) a recoiling lithium (^7^Li) nucleus.^9^ These particles generate lethal ionization over a short trajectory (<10 μm), closely aligned with the diameter of a single cell (~10 μm),^1^ inducing the generation of reactive oxygen species (ROS), DNA double‐strand breaks, and eventually cell death. Sodium mercaptoundecahydrododecaborate (BSH) and L‐*p*‐boronophenylalanine (BPA) are commonly used as boron‐delivery agents, but their effectiveness in clinical BNCT remains suboptimal, necessitating further improvement. This limitation stems from limited aqueous solubility (particularly BPA) and the fact that they rely on limited transport mechanisms, resulting in inadequate boron‐delivery capacity to the tumor site.^10^

**FIGURE 1:**
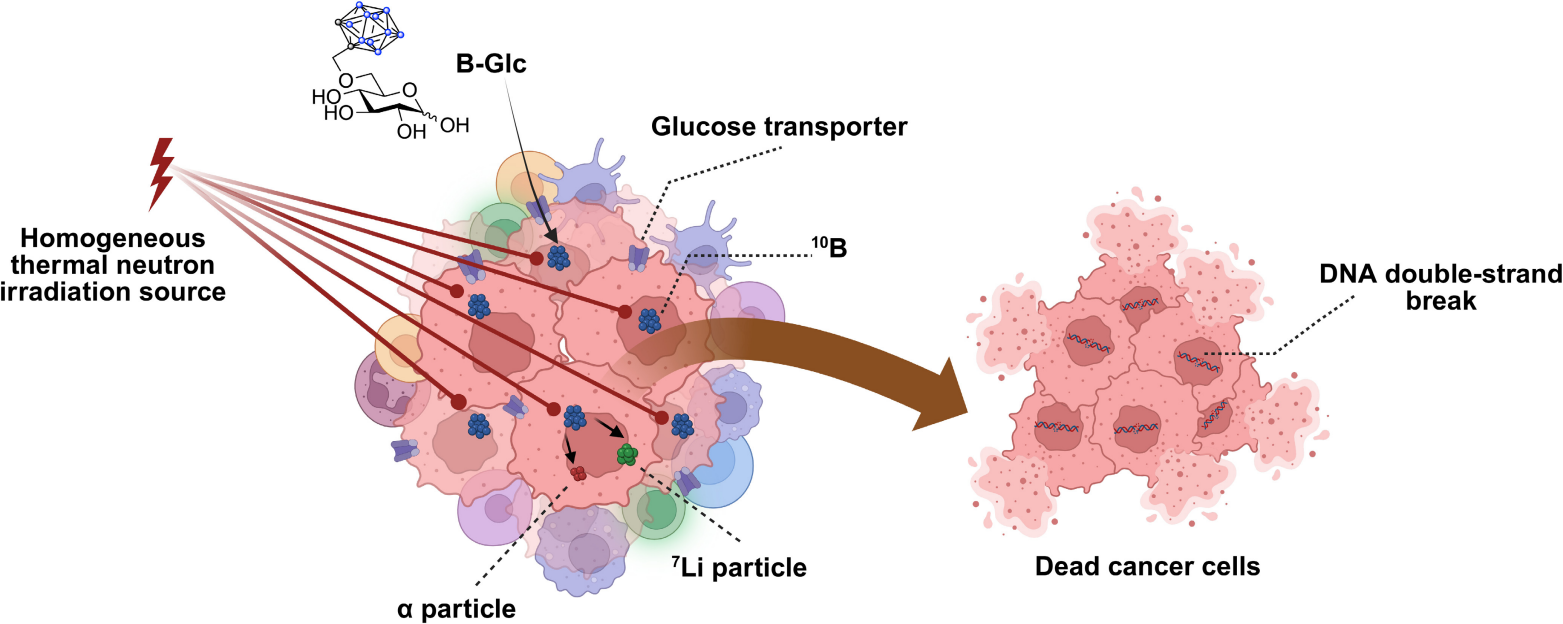
Targeted delivery of the B‐Glc via the glucose transporter GLUT1, followed by tumor irradiation with a homogeneous thermal neutron beam. The irradiation is specifically focused and uniformly distributed within the tumor area, inducing the emission of α and ^7^Li particles. This results in DNA double‐strand breaks and eventual cancer cell death, while sparing surrounding healthy cells. Figure created using https://BioRender.com/w46g324.

To this end, significant effort has been made to improve targeted therapy for BNCT,^11^ with the goal of rendering boron‐delivery carriers more selective to biological targets expressed on tumor cells while optimizing in vivo pharmacokinetics and minimizing systemic toxicity.^12^, ^13^, ^14^, ^15^ Glucose transporters (GLUTs), a family of transmembrane proteins, play a significant role in glucose uptake, facilitating glycolysis and adenosine triphosphate (ATP) production in mammalian cells.^16^ Tumor cells are known to have an elevated glucose consumption due to a rapid growth,^17^ primarily through the abnormal upregulation of glucose transporters, particularly glucose transporter 1 (GLUT1).^18^ Overexpression of GLUT1 across various cancers^19^ has positioned it as a pan‐tumor target for cancer imaging and therapy.^20^, ^21^, ^22^ In HNSCC, previous reports have demonstrated high GLUT1 expression, while other glucose transporters such as GLUT3 are less extensively studied and less consistently expressed. Other GLUT isoforms, including GLUTs 2, 4, 8, and 13, are generally undetectable in HNSCC tissues, while GLUTs 7 and 14 have not been investigated to date.^23^, ^24^, ^25^, ^26^, ^27^ In recent years, our focus has centered around synthesizing and characterizing a series of glucoconjugates bearing boron clusters and investigating their biochemical foundations of GLUT1 targeted approach to BNCT in HNSCC cell models (Figure 1). In general, the glucoconjugate boron carriers have exhibited notable attributes, including adequate aqueous solubility, good cytocompatibility, and a robust binding affinity to GLUT1 in human HNSCC CAL 27 cells in vitro.^28^, ^29^, ^30^, ^31^ Additionally, in vitro cell uptake studies revealed that these glucoconjugates deliver substantial amounts of boron to the cells without disrupting normal glucose metabolism. This suggests that they are not metabolized, allowing for increased intracellular retention and concentration of boron into cancer cells. Overall, results from both in vitro experimental and computational approaches have demonstrated that the 6‐*O*‐(*o*‐carboranylmethyl)‐d‐glucopyranose (referred to B‐Glc from herein) displays promising properties compared to the current clinically used boron delivery agents like BSH and BPA.

This study aims to further investigate the in vivo pharmacokinetics of the B‐Glc to ascertain its viability as a novel boron‐delivery agent for BNCT. Our investigation dived into its biological behavior in several human HNSCC cell models, including CAL 27, UT‐SCC‐14, UT‐SCC‐28, and UT‐SCC‐42B cells, as well as animals bearing CAL 27 and UT‐SCC‐14 tumor xenografts. Additionally, we employed diagnostic positron emission tomography (PET) supplemented with computed tomography (CT) imaging to characterize and validate GLUT1 expression of CAL 27 and UT‐SCC‐14 tumors, utilizing the gold standard [^18^F]fluoro‐2‐deoxy‐d‐glucose ([^18^F]FDG) radiotracer for glucose metabolism imaging. Biodistribution studies in mice with CAL 27 and UT‐SCC‐14 orthotopic xenografts in the tongue were conducted, as well as western blot, histological, and immunohistochemical analyses to preliminarily assess the potential clinical translation of the B‐Glc.

## 2 MATERIALS AND METHODS

### 2.1 Chemicals and reagents

Unless specified otherwise, all chemicals and organic solvents were procured from commercial vendors as indicated and used without additional purification.

### 2.2 Boron carriers

The synthesis and characterization of the 6‐*O*‐(*o*‐carboranylmethyl)‐d‐glucopyranose (B‐Glc) utilized in this study are detailed in a prior report.^28^ The sodium mercaptoundecahydrododecaborate (BSH) was acquired from Kat‐Chem Kft. (Budapest, Hungary). The L‐*p*‐boronophenylalanine fructose complex (BPA‐F) solution was prepared at the Helsinki University Hospital Pharmacy according to the *European Pharmacopoeia* (30 g/l L‐BPA and 10% molar excess of fructose in sterile isotonic saline) and used as received.

### 2.3 Cell lines

The human University of Turku–Squamous Cell Carcinoma cell lines, including UT‐SCC‐14 (RRID: CVCL_7810), UT‐SCC‐28 (RRID: CVCL_7832), and UT‐SCC‐42B (RRID: CVCL_7848), were kindly provided by Professor Reidar Grènman at the Department of Otorhinolaryngology – Head and Neck Surgery, Turku University Hospital, University of Turku, Turku, Finland. The human squamous cell carcinoma CAL 27 (CRL‐2095™, RRID: CVCL_1107) and keratinocyte CCD 1106 KERTr (CRL‐2309™, RRID: CVCL_3300) cell lines were acquired from the American Type Culture Collection (ATCC, Manassas, VA, USA). These cell lines were used throughout the in vitro and in vivo evaluation, representing HNSCC and relevant healthy cell models. All experiments were performed with mycoplasma‐free cells, confirmed to be so using the MycoAlert PLUS kit (Lonza, Basel, Switzerland). DNA profiles were extracted from each cell line using the GenePrint® 24 System (Madison, WI) for genotyping to verify cell line identity, and the human cell lines have been authenticated for identity using STR profiling within the last 3 years. Details regarding cell culture reagents and general procedures are described in the Data S1.

### 2.4 Cell cytotoxicity

Cells were seeded on an opaque 96‐well plate at a density of 5 × 10^3^ cells per well and allowed to attach overnight. The medium was then replaced with complete cell culture medium containing either the B‐Glc or BSH at concentrations ranging from 5 to 250 μM. The negative control included 0.1% DMSO in complete cell culture medium, while a 1% (v/v) Triton X‐100 solution served as the positive control. At designated time points (6, 24, and 48 h), cell viability was assessed using the CellTiter‐Glo® luminescent cell viability assay (Promega, Madison, WI), following the manufacturer's protocol. Luminescence was measured using the VICTOR Nivo™ multimode plate reader (Perkin Elmer Inc., Waltham, MA). All measurements were conducted in triplicate.

### 2.5 GLUT1 binding affinity and cellular uptake

Cells were seeded onto a 24‐well plate at a density of 5 × 10^5^ cells per well and allowed to adhere for 24 h. The incubation medium was then replaced with warm glucose‐free 1 × HBSS (pH 7.4) to pre‐incubate the cells at 37°C for 10 min before starting subsequent experiments.

For binding affinity studies, the cells were incubated at room temperature (RT) for 5 min with various concentrations of the B‐Glc (0.1–1800 μM) in 250 μL of glucose‐free 1 × HBSS (pH 7.4) containing [^14^C]‐d‐glucose (1.8 μM, 0.1 mCi/mL, PerkinElmer Inc., Waltham, MA). The incubation was terminated by adding ice‐cold 1 × HBSS, followed by two washes with ice‐cold 1 × HBSS. The cells were then lysed with 250 μL of 0.1 M sodium hydroxide, mixed with 1 mL of Emulsifier Safe Cocktail (PerkinElmer Inc., Waltham, MA), and analyzed by liquid scintillation measurement using the MicroBeta 2 counter (PerkinElmer Inc., Waltham, MA). The half‐maximal inhibitory concentration (IC_50_) was determined using nonlinear regression analysis.

For cellular uptake studies, the B‐Glc (10–400 μM) in 250 μL of glucose‐free 1 × HBSS (pH 7.4) was added to the cells and incubated for 30 min at RT (*n* = 4). Cells were then washed and lysed as described above. Lysates from four identical wells were combined and centrifuged at 4°C, 1200 rpm. The supernatant (800 μL) was collected and digested with 1 mL of concentrated nitric acid at 4°C for 24 h. The final volume was adjusted to 10 mL with ultrapure water (Purelab Ultra, ELGA LabWater, Lane End, UK) before boron analysis using inductively coupled plasma mass spectrometry (NeXION 350D ICP‐MS, PerkinElmer Inc., Waltham, MA), equipped with an electrospray ionization (ESI) PrepFAST autosampler (Elemental Scientific, Omaha, NE), with all measurements conducted in triplicate. Detailed instrument settings are provided in our previous report.^28^ Data processing was carried out using PerkinElmer Syngistix Data Analysis software.

### 2.6 Western blotting and proteomics

Western blotting and targeted proteomic analysis (for GLUT1) by mass spectrometry of cell lysates were conducted to assess GLUT1 expression levels in the studied cell lines. Detailed procedures are provided in the Data S1.

### 2.7 HNSCC orthotopic xenograft models

Detailed information about the animals and housing conditions is provided in the Data S1. For the orthotopic sublingual tumor model, 1 × 10^5^ CAL 27 or UT‐SCC‐14 cells (viability >96%) in 25 μL of sterile 1 × DPBS (pH 7.4) were injected into the right side of the tongue under anesthesia, using a mixture of medetomidine (0.5 mg/kg, Domitor®, Orion Pharma, Espoo, Finland) and ketamine (50 mg/kg, Ketaminol® Vet., MSD Animal Health, Espoo, Finland) in 100 μL of sterile isotonic saline administered subcutaneously. Following inoculation, atipamezole (0.5 mg/kg, Revertor® Vet., Vet Medic Animal Health, Parola, Finland) in 100 μL of sterile isotonic saline was injected subcutaneously to reverse the effect of the anesthesia. Post‐procedural care included perioperative carprofen (5 mg/kg, Rimadyl®, Zoetis, Helsinki, Finland) in 100 μL of sterile isotonic saline and warm sterile isotonic saline (200 μL) administered subcutaneously. Animal weight and physical conditions were monitored daily, and tumor size was assessed once a week under 2.5% isoflurane anesthesia in air and medical oxygen gas mixture (60:40, 1 L min^−1^). The tumor was allowed to develop to a size of at least 3–4 mm in the largest dimension (approximately 2 weeks after tumor implantation) before the start of experiments. Furthermore, untreated CAL 27 and UT‐SCC‐14 tumors were collected for immunohistochemical analysis to assess GLUT1 expression. The procedures are available in the Data S1.

### 2.8 PET/CT imaging

To assess functional glucose uptake in vivo, PET/CT imaging was carried out in both CAL 27 and UT‐SCC‐14 tumor‐bearing animals under 3‐h or 6‐h fasting and non‐fasting conditions, using [^18^F]FDG radiotracer obtained either from a clinical routine production at Turku PET Centre, Turku University Hospital, or the Cyclotron Unit, Helsinki University Hospital. [^18^F]FDG radiotracer was administered intravenously to the animals via a lateral tail vein at an average activity dose of 2.92 ± 0.17 (~370 GBq/μmol, Turku University Hospital production) and 11.69 ± 0.26 MBq (~1447 GBq/μmol, Helsinki University Hospital production) in 60–100 μL of sterile isotonic saline. The whole‐body PET/CT imaging was acquired for a 90‐min dynamic scan or 20‐min static scan after 60‐min post‐injection (p.i.) under 1.5–3% isoflurane anesthesia in medical oxygen carrier (1 L min^−1^) using the Molecubes benchtop β‐CUBE and X‐CUBE (Molecubes NV, Gent, Belgium) for PET and micro‐CT, respectively. A helical CT scan was firstly conducted with a 50 kVp X‐ray source followed by a PET imaging. At the end of the scan, animals were euthanized, and tissues of interest were collected for gamma counting. The image analysis was done using the VivoQuant™ 2021 software (InviCRO LLC, Needham, MA, USA). The results are presented as the standardized uptake value (SUV) for PET images and percent of injected dose per gram of tissue (%ID/g) for biodistribution with gamma counting.

### 2.9 Biodistribution

Animals were randomly grouped for each cohort of experiments, comprising fasting and non‐fasting groups (*n* = 3–4). The fasting condition was introduced to specifically investigate the effect of blood glucose competition; both the pelleted food and diet gel were removed from the cage at the end of the 12‐h dark cycle for 6 h before treatments. The B‐Glc formulation was prepared in sterile 1× PBS (pH 7.4) supplemented with 5% (v/v) ethanol and 5% (v/v) nonionic solubilizer (Solutol® HS 15, Merck, Rahway, NJ, USA), while BPA was formulated in a fructose complex (BPA‐F) solution as described above. The intravenous administration of the B‐Glc formulations (200 μL) was done via the lateral tail vein at a dose of 50–100 mg/kg, whereas BPA‐F was injected as a bolus of 400 mg/kg (clinically relevant dose). Animals were euthanized by CO_2_ asphyxiation at predetermined time points (5, 15, 30, and 60 min), and relevant tissues were collected, including urine, blood, gallbladder, liver, kidney, muscle, tumor, tongue (healthy tissue), oral mucous membrane, and brain. Boron accumulation in tissue samples was quantified using microwave plasma atomic emission spectroscopy (MP‐AES 4200, Agilent Technologies, Santa Clara, CA, USA). The sample preparation for MP‐AES measurement is detailed in the Data S1.

### 2.10 Statistical analysis

Quantitative data are expressed as mean ± SD (*n* ≥ 3). Direct comparison between sample groups was carried out using unpaired Student's *t*‐tests with unequal variances, without correction for multiple comparisons. Statistical significance (*p*‐value) thresholds were set at *p < 0.05, **p < 0.01, ***p < 0.001, and n.s. denoting not significant. GraphPad Prism software versions 5.03 and 10 (San Diego, CA) was used for nonlinear regression, statistical analysis, and graphical representation.

## 3 RESULTS AND DISCUSSION

As part of our ongoing efforts to develop innovative boron‐delivery agents for BNCT, we conducted further investigations into one of the promising candidates from our previous study—the 6‐*O*‐(*o*‐carboranylmethyl)‐d‐glucopyranose or B‐Glc.^28^ The in vitro investigation was broadened to include three additional patient‐derived HNSCC cell lines: UT‐SCC‐14, UT‐SCC‐28, and UT‐SCC‐42B, originating from the tongue, oral cavity, and larynx, respectively. The goal of these studies was to identify the most appropriate cell line for subsequent in vivo investigations by comparing cytotoxicity, binding affinity, and cellular uptake of the B‐Glc across these cell lines.

### 3.1 In vitro cytotoxicity

The cytotoxicity assay conducted herein served as an initial screening to assess the potential toxicity and subcellular targeting of the glucose‐based boron carrier, aiming to provide insights into its possible in vivo effects. In this study, we compared the toxicity of the B‐Glc with the clinically relevant boron delivery agent BSH in several human‐derived HNSCC cell lines, including UT‐SCC‐14, UT‐SCC‐28, and UT‐SCC‐42B, as well as the keratinocyte CCD 1106 cell line, used as a tissue‐relevant control. Overall, the B‐Glc exhibited no significant cytotoxicity at concentrations up to 125 μM across all time points (Figure S1). However, a marked reduction in cell viability was observed at the highest concentration (250 μM) after 24 h of incubation in all tested cell lines. In contrast, BSH demonstrated no notable cytotoxic effects in the HNSCC cell lines at any tested concentration or time point, with cell viability consistently remaining above approximately 80% after 48 h of incubation. These results suggest that the cytotoxic effects of the B‐Glc at higher concentrations (125–250 μM) might be more pronounced due to its enhanced uptake through GLUT1 transporters, a mechanism not observed with BSH uptake. Moreover, the observed reduction in CCD1106 cell viability at high B‐Glc concentrations, despite lower GLUT1 expression, may be due to their increased sensitivity to boron‐induced cytotoxicity, potentially linked to differences in oxidative stress response or DNA damage repair. Additionally, metabolic differences between CCD1106 and HNSCC cells could influence B‐Glc processing, with lower metabolic activity in CCD1106 cells potentially making them more vulnerable to boron toxicity imparted by B‐Glc in the glycolysis pathway. Overall, these findings align with our previous cytotoxicity results for this glucoconjugate in the CAL 27 cell line,^28^ further supporting its promising cytocompatibility and potential as an effective boron delivery agent for BNCT.

### 3.2 GLUT1 binding affinity and in vitro cell uptake

The ability of the B‐Glc to compete with the known substrate d‐glucose (1.8 μM) for GLUT1 binding was assessed in all three HNSCC cell lines. The IC_50_ values of the B‐Glc, determined from the *cis*‐inhibition assay, were 93.92 μM in UT‐SCC‐14, 190 μM in UT‐SCC‐28, and 258 μM in UT‐SCC‐42B cells, indicating varying affinities across the cell lines, with the highest potency observed in UT‐SCC‐14 cells. While the affinity studies only demonstrate the ability of the B‐Glc to compete with d‐glucose for GLUT1 binding, we also conducted cell uptake studies following 30 min of incubation to further assess its cellular internalization.

Among the tested HNSCC cell lines, the B‐Glc showed particularly notable Michaelis–Menten kinetic parameters—*V*_max_ (maximum velocity) and *K*_*m*_ (Michaelis constant)—in uptake studies in UT‐SCC‐14 cells compared to the other two cell lines (Table 1 and Figure S2).

**TABLE 1:** Michaelis–Menten kinetic parameters (*V*_max_ and *K*_*m*_ values in μM) of the B‐Glc, BPA, and BSH determined in uptake studies conducted in UT‐SCC‐14, UT‐SCC‐28, and UT‐SCC‐42 B cell lines following 30 min of incubation.

Boronated agent	UT‐SCC‐14		UT‐SCC‐28		UT‐SCC‐42B	
	*V*_max_ (μM)	*K*_*m*_ (μM)	*V*_max_ (μM)	*K*_*m*_ (μM)	*V*_max_ (μM)	*K*_*m*_ (μM)
B‐Glc	10.80 ± 3.00	351.6 ± 167.9	26.29 ± 5.51	1078 ± 287.3	14.42 ± 1.10	511.9 ± 59.9
BPA	0.97 ± 0.27	439.2 ± 197.1	N/D	N/D	2.01 ± 1.10	1629 ± 1053
BSH	0.15 ± 0.06	588.4 ± 357.1	N/D	N/D	N/D	N/D

*Note*: “N/D” indicates not detected.

The B‐Glc exhibited the highest affinity in UT‐SCC‐14 (*K*_*m*_ 351.6 ± 167.9 μM), followed by UT‐SCC‐42B (*K*_*m*_ 511.9 ± 59.9 μM) and UT‐SCC‐28 (*K*_*m*_ 1078 ± 287.3 μM) cells. In UT‐SCC‐14 cells, the *V*_max_ of the B‐Glc (10.8 ± 3.00 μM) was 10‐fold higher than that of BPA (0.97 ± 0.27 μM) and 72‐fold higher than BSH (0.15 ± 0.06 μM). Similarly, in UT‐SCC‐42B cells, the *V*_max_ of the B‐Glc (14.42 ± 1.1 μM) was 7 times greater than that of BPA (2.01 ± 1.1 μM), while BSH uptake remained negligible. For UT‐SCC‐28 cells, the uptake of BPA and BSH was insignificant, precluding the calculation of Michaelis–Menten parameters; however, the *V*_max_ of the B‐Glc in UT‐SCC‐28 cells was the highest among the three (26.29 ± 5.51 μM). This high uptake in UT‐SCC‐28 cells aligns with proteomics data, which identified this cell line as having the highest GLUT1 expression (Figure S3A). Additionally, western blot analysis, consistent with proteomics data, revealed elevated GLUT1 expression in all HNSCC cell lines used in this study compared to the keratinocyte CCD 1106 cell line, which served as a control for GLUT1 expression in normal cells (Figure S3B). In general, these in vitro studies revealed that the B‐Glc demonstrated significantly superior uptake compared to currently available boron delivery agents in clinical use. Based on overall cytotoxicity results, binding affinity, uptake profiles, and GLUT1 expression, the UT‐SCC‐14 cell line was chosen for further in vivo studies.

### 3.3 PET/CT imaging

GLUT1 expression has been found to be upregulated in patients with HNSCC, serving as a prognostic marker for predicting the clinical stage of the disease.^32^ This upregulation has a direct link to the malignant transformation process, marked by an increase in glucose utilization within the tumor.^33^ As GLUT1 is the target of the B‐Glc in this study, [^18^F]FDG radiotracer was employed as a surrogate to observe glucose uptake in both CAL 27 and UT‐SCC‐14 tumors in vivo. The [^18^F]FDG enters cells and is phosphorylated into [^18^F]FDG‐6‐phosphate, which stalls further metabolism and residualizes the radiotracer intracellularly, with the rate of this process directly proportional to tissue glucose uptake. This allows for the straightforward visualization of glucose uptake via the glucose transporters in various tissues throughout the body based on the radioactive signal. Moreover, the studied B‐Glc is not a substrate for glucose metabolism either,^34^ potentially rendering [^18^F]FDG PET imaging a diagnostic tool for patient selection in GLUT1 targeted BNCT.

In our initial imaging experiment, we conducted a 90‐min dynamic PET/CT scan of [^18^F]FDG (~3 MBq bolus injection per animal, ~48 ng of [^18^F]FDG) in UT‐SCC‐14 tumor‐bearing mice following a 3‐h fasting period at different stages of tumor growth (6, 11, and 13 days after tumor implantation). Notably, the dynamic PET/CT images failed to clearly delineate [^18^F]FDG accumulation in the tumors, despite immunohistochemical analysis showing substantial GLUT1 expression in the UT‐SCC‐14 tumor (Figure S4A, right panel). Furthermore, no significant differences in uptake patterns were observed across the time points, with major uptake occurring only in the harderian glands, the myocardium (rich in GLUT1), and in the urinary bladder where [^18^F]FDG is excreted (Figure S5). These findings align with a report in the literature on how animal handling, including fasted versus fed state, ambient temperature, and use of anesthesia, can affect [^18^F]FDG biodistribution in PET studies in tumor‐bearing mice.^35^ Although the animals were fasted (3 h), kept warm, and anesthetized during the [^18^F]FDG administration and uptake periods—conditions that should ideally minimize [^18^F]FDG accumulation in background tissues—we suspect that the inactive state of animals under anesthesia during the 90‐min dynamic PET scan may have significantly favored myocardial uptake of [^18^F]FDG over tumor uptake in this model.

**FIGURE 2:**
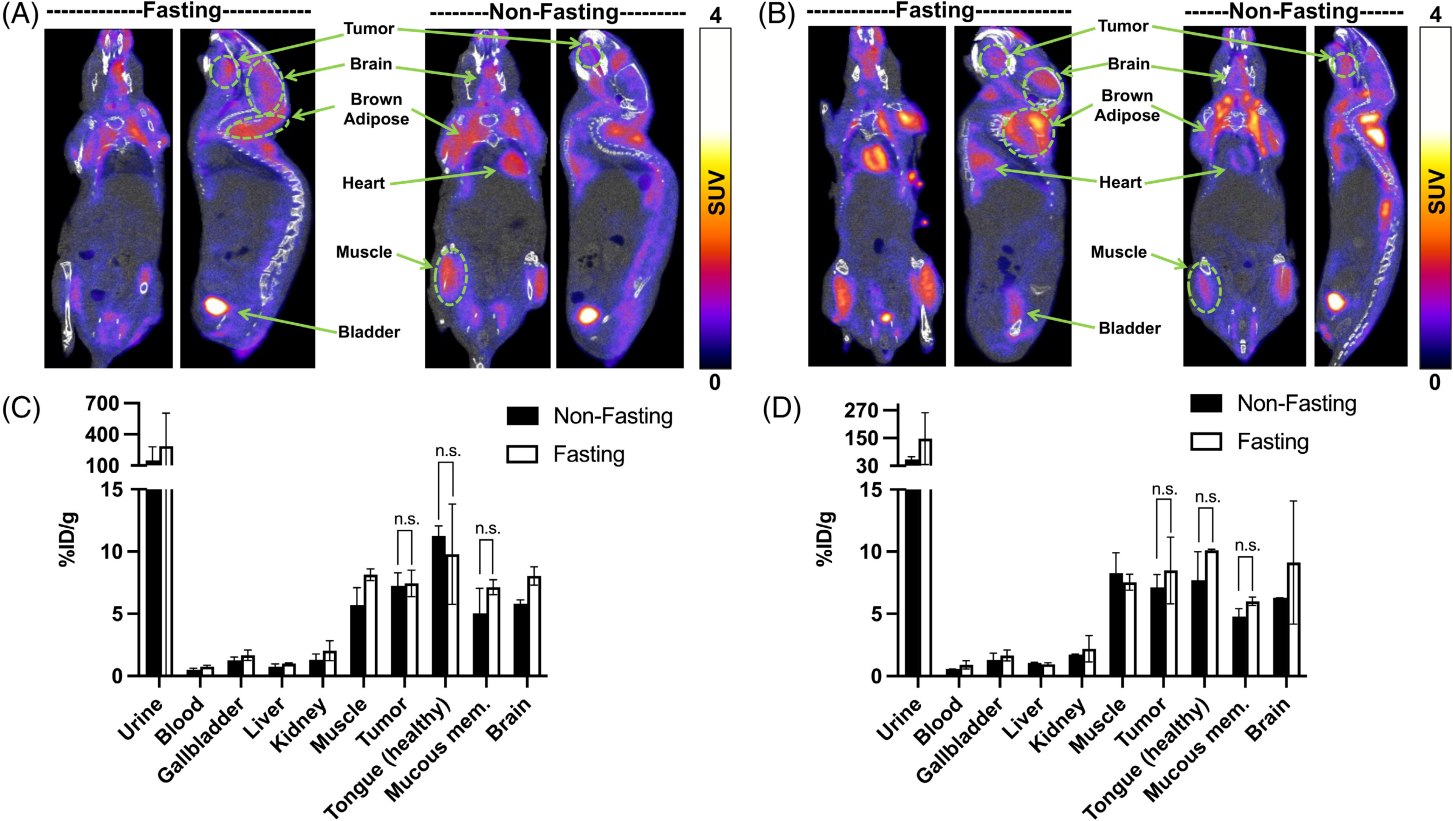
Representative static PET/CT images in coronal (left) and sagittal (right) planes in fasting and non‐fasting conditions of (A) CAL 27 and (B) UT‐SCC‐14 tumor‐bearing mice captured at 60 min after [^18^F]FDG administration at 14–15 days of tumor implantation, and subsequent biodistribution profiles of (C) CAL 27 and (D) UT‐SCC‐14 tumor‐bearing animals after PET/CT imaging. The presented values indicate the mean ± SD (*n* = 3) where statistical significance was set at *p* < 0.05 and ns, not significant. The %ID/g denotes percent injected dose per gram of tissue and mucous mem. for mucous membrane.

In our second attempt, we optimized animal handling conditions to enhance the delineation and visualization of [^18^F]FDG tumor uptake. We extended the fasting period to 6 h, warmed the animals for at least 30 min before [^18^F]FDG administration, and kept them conscious during the tracer injection until imaging. Subsequently, we conducted a 20‐min static PET/CT imaging after 60 min of [^18^F]FDG administration using approximately 11 MBq (~25 ng of [^18^F]FDG) bolus injection per animal on CAL 27 and UT‐SCC‐14 tumor‐bearing mice at 14–15 days after tumor implantation. In general, PET/CT showed that [^18^F]FDG was predominantly cleared through the kidneys within 60 min p.i., with high uptake in harderian glands, brain, brown adipose tissue, heart, skeletal muscle, and this time also in the tumor in both tumor models (Figure 2A for CAL 27 and 2B for UT‐SCC‐14). This observation aligns with the previously reported high accumulation of [^18^F]FDG in these tissues in conscious animals.^36^, ^37^ Surprisingly, while we achieved better tumor delineation and visualization in the second attempt, there was no significant difference (*p* > 0.05) in [^18^F]FDG tumor uptake between fasting and non‐fasting groups in both CAL 27 and UT‐SCC‐14 tumor models. This was also confirmed by biodistribution results (Figure 2C for CAL 27, 2D for UT‐SCC‐14) and the tumor SUV values obtained from PET/CT image analysis (Table S1).

In general, PET/CT imaging and biodistribution demonstrated active glucose uptake in the tumor, but as can be seen from the biodistribution data, the surrounding healthy tongue tissue also has [^18^F]FDG uptake when injected to awake mice, likely due to muscle activity, such as licking and chewing, occurring prior to the scan. Nevertheless, immunohistochemical analysis confirmed GLUT1 expression in both tumors at a higher degree relative to the surrounding tissue, with UT‐SCC‐14 exhibiting membrane localization of the transporter (Figure S4A). Moreover, hematoxylin and eosin (H&E) staining clearly delineated tumor masses in the tongues of mice bearing CAL 27 and UT‐SCC‐14 xenografts (Figure S4B), while the specificity of GLUT1 primary antibody binding in immunohistochemical analysis was confirmed using a rabbit IgG isotype control (Figure S4C). Altogether, PET/CT imaging combined with immunohistochemical analysis corroborates the feasibility of using [^18^F]FDG as a companion diagnostic for B‐Glc in HNSCC, but the animal handling and imaging protocols warrant further optimization. It is highly likely this is not a major issue in human patients, who are asked to avoid strenuous exercise for 24 h, fast for 4 h, and remain still and silent prior to the [^18^F]FDG PET study to avoid muscle uptake of the radiotracer.^38^

### 3.4 Biodistribution studies

In the first cohort, the biodistribution was assessed in non‐fasting orthotopic CAL 27 tumor‐bearing mice as the first step in examining the optimal dosage and clearance profiles of the B‐Glc in vivo at 60 min p.i., aligning with the timeframe of BPA‐F intravenous infusion in clinical BNCT.^39^ The studied doses of the B‐Glc were set at 50 and 100 mg/kg. The biodistribution profiles in CAL 27 tumor‐bearing mice are shown in the Figure S6. Briefly, major uptake was seen in the liver, with an average total boron concentration of 77 ppm for 50 mg/kg and 150 ppm for 100 mg/kg, as anticipated due to its role as the primary organ responsible for drug metabolism in the body. Aside from hepatobiliary elimination, renal clearance was also found to be a primary pathway for eliminating the administered B‐Glc from the circulation, as evidenced by boron excretion in the gallbladder, kidney, and urine with increasing dosage. Unexpectedly, increasing the dose did not enhance tumor uptake (21 ppm for 50 mg/kg and 13 ppm for 100 mg/kg, respectively). In terms of safety, the 50 mg/kg dose exhibited no acute toxicity or severe side effects during biodistribution studies; however, animals administered the 100 mg/kg dose displayed visible hematuria. Hence, the biodistribution profile and overall health status of the animals in this initial cohort with a limited animal number warrant further investigation of the B‐Glc biodistribution at different time points to obtain a more comprehensive pharmacokinetics and safety profile. It is also advisable to consider a maximum dose below 100 mg/kg in subsequent studies to prevent overdosing that can pose a significant risk to animal health and experimental outcomes without improvement in the tumor uptake.

**FIGURE 3:**
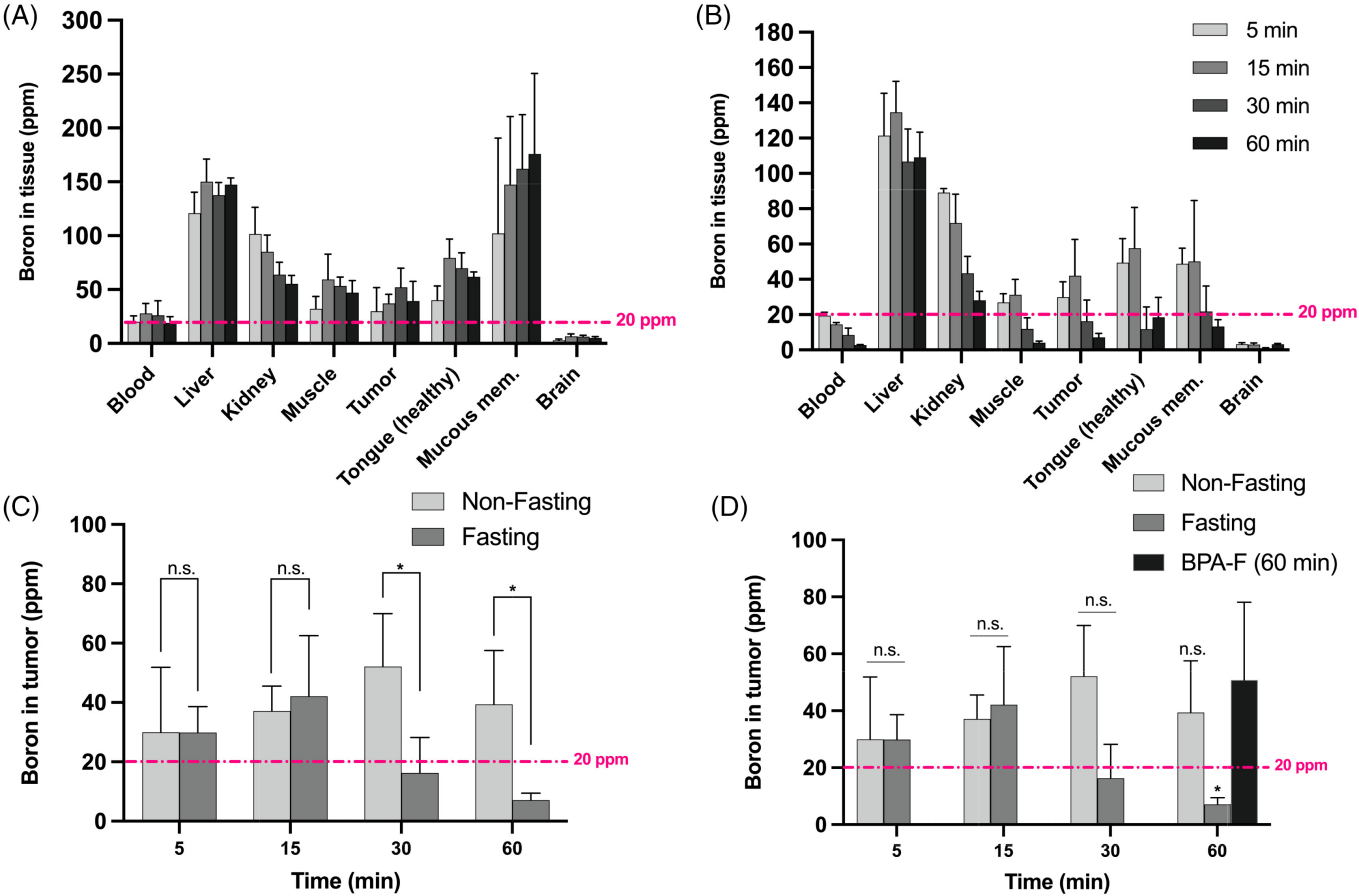
Biodistribution profiles following B‐Glc administration at 75 mg/kg in UT‐SCC‐14 tumor‐bearing mice under (A) non‐fasting and (B) fasting conditions at 5, 15, 30, and 60 min. (C) Comparative analysis of B‐Glc tumor uptake between non‐fasting and fasting groups. (D) Comparative analysis of tumor uptake in B‐Glc groups to clinically relevant BPA‐F (400 mg/kg) group. The presented values indicate the mean ± SD (*n* = 3–4). Statistical significance was set at *p < 0.05, and n.s., not significant. The ppm denotes parts per million unit and mucous mem. for the mucous membrane. The dashed line in pink represents the 20‐ppm cutoff, which is the minimum ^10^B concentration considered effective for BNCT.

In a following set of biodistribution studies, we opted to use the UT‐SCC‐14 tumor model sourced from patient‐derived oral tongue cancer at Turku University Hospital, Turku, Finland, to have a potentially clinically more relevant model for our investigation. To assess the effect of competition by plasma glucose levels on the B‐Glc uptake, animals bearing UT‐SCC‐14 tumor xenografts were randomized into two groups, fasting and non‐fasting, before the treatment. The dose of the B‐Glc in this study was fixed at 75 mg/kg based on the outcome from biodistribution studies in CAL 27 tumor‐bearing mice earlier. Animals in each group were euthanized at 5, 15, 30, and 60 min after treatment, and tissue samples were collected for boron measurement. In general, there is no difference in the biodistribution profile between non‐fasting (Figure 3A) and fasting (Figure 3B) groups with major uptake in the liver and kidney, along with renal clearance (Figure S7A,B). Tumor uptake increased over time in both animal groups (Figure 3C), with the highest uptake (52 ppm) found at 30 min for the non‐fasting group and at 15 min (42 ppm) for the fasting group. However, while there is no significant difference in tumor uptake between the two animal groups at 15 min, a difference can be seen at 30 min p.i. (*p* < 0.05). This indicates that plasma glucose levels might not significantly affect tumor uptake of the B‐Glc. Interestingly, both groups showed a significant decrease in tumor uptake at 60 min p.i., which could be attributed to the effect of glucose turnover rate; however, monitoring metabolic fluxes of the B‐Glc in glycolysis may pose challenges due to the limited availability of suitable methods.^40^

Furthermore, we carried out a small cohort biodistribution study of BPA‐F in the fasting group with a 400 mg/kg injected dose and a 60‐min lag time to generate a baseline dataset for comparison with the B‐Glc. While BPA‐F generally exhibited comparable pharmacokinetics to the B‐Glc (Figure S8), it appeared to surpass the latter in terms of tumor uptake (51 ppm on average), as opposed to 39 ppm for the non‐fasting (*p*‐value: n.s.) and 7 ppm for the fasting groups (*p* < 0.05) receiving B‐Glc at 60‐min p.i. This difference may be attributed to the boron cluster modification on d‐glucose, which could have altered the in vivo pharmacokinetics of B‐Glc, resulting in rapid systemic clearance and unsatisfactory tumor accumulation relative to BPA‐F. Typically, a minimum concentration of 20 ppm of ^10^B in the tumor is required to achieve a therapeutic effect with boron neutron capture.^41^ With this in mind, the performance of B‐Glc in the non‐fasting group at all time points and the fasting group at 5–30 min is comparable to that of BPA‐F at 60 min (*p*‐value: n.s.), all demonstrating an average concentration of more than 20 ppm of ^10^B in the tumor (Figure 3D).

**FIGURE 4:**
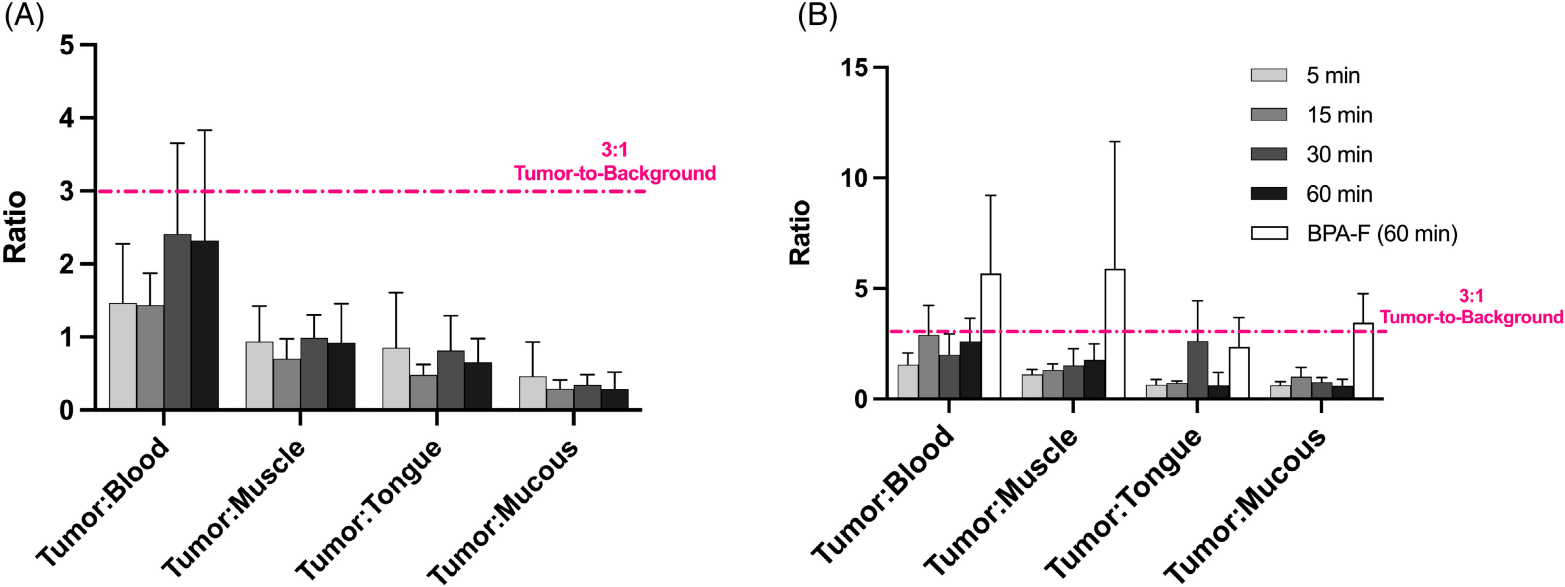
Tumor‐to‐background ratios of the B‐Glc (75 mg/kg) in UT‐SCC‐14 tumor‐bearing animals in (A) non‐fasting and (B) fasting animal groups at 5, 15, 30, and 60 min and BPA‐F (400 mg/kg, investigated only in the fasting group) at 60 min. The values represent the mean ± SD (*n* = 3–4). Mucous indicates oral mucous membrane.

Regarding off‐target accumulation, BPA‐F at the 60‐min time point outperformed the B‐Glc, both at the 30 min in the non‐fasting group (Figure 4A) and 15 min in the fasting group (Figure 4B), where the highest boron uptake in the tumor for B‐Glc was observed. BPA‐F achieved at least 2‐fold higher tumor‐to‐background ratios compared to the B‐Glc across all relevant background tissues, including blood, muscle, healthy tongue, and oral mucous membranes. The tumor‐to‐background ratio of the B‐Glc in all time points generally remained suboptimal, falling short of the ideal threshold of a 3:1 tumor‐to‐background ratio in tissues that would be exposed to neutron irradiation.^42^ Nevertheless, the in vivo performance of the B‐Glc illustrates substantial tumor uptake exceeding 20 ppm faster after administration and with a 5‐fold lower injected dose (75 mg/kg) compared to BPA‐F (400 mg/kg), showing promise as an alternative boron carrier for BNCT. However, the optimal timing of the neutron irradiation relative to the B‐Glc administration and the effect of repeated dosing remain to be investigated to achieve the improved tumor‐to‐background ratios.

Among targeted boron delivery strategies, glucose‐based boron carriers such as B‐Glc offer a metabolically driven alternative to ligand‐targeted approaches. While peptide‐based systems such as RGD peptides targeting α_v_β_3_ and α_v_β_5_ integrins have demonstrated tumor specificity, particularly in glioblastoma, they could face limitations in terms of in vivo stability, boron carrying capacity, tumor penetration, and pharmacokinetics. In contrast, B‐Glc exploits the elevated glucose uptake commonly observed in tumors and is already harnessed successfully for tumor imaging with [^18^F]FDG PET/CT, offering a broader tumor‐targeting potential. Moreover, B‐Glc presents a modular platform that could be further enhanced through conjugation with other targeting moieties, such as peptides, or by incorporation into nanoparticle‐based delivery systems. Such strategies could improve tumor selectivity and boron delivery efficacy for BNCT, broadening the therapeutic potential of glucose‐based boron carriers.

## 4 CONCLUSIONS

This study systematically assessed the in vivo performance of a GLUT1‐targeting boron carrier based on glucose through biodistribution studies in CAL 27 and UT‐SCC‐14 tumor xenografts, aiming to evaluate its potential as an alternative boron carrier for BNCT. PET/CT imaging, alongside protein expression and immunohistochemical analyses, confirmed effective GLUT1 targeting in these tumor models. Biodistribution results indicated that the optimal dosage and lag time before neutron irradiation should be below 100 mg/kg and 60 min, respectively. Moreover, the B‐Glc demonstrated rapid pharmacokinetics, exhibiting satisfactory tumor uptake in a shorter timeframe compared to clinically employed BPA‐F. Despite meeting some of the expectations in in vivo performance for a boron carrier for BNCT, the B‐Glc fell short of achieving the optimal 3:1 tumor‐to‐background ratios, emphasizing the need for further optimization of compound pharmacokinetics and dosing regimen. Overall, these findings warrant additional investigations into the therapeutic potential of the B‐Glc for BNCT and illustrate the potential of using [^18^F]FDG PET in patient selection for BNCT with GLUT1‐targeted boron delivery agents.

## AUTHOR CONTRIBUTIONS

**Surachet Imlimthan:** Conceptualization; methodology; data curation; investigation; validation; formal analysis; funding acquisition; visualization; writing – original draft; writing – review and editing. **Katayun Bahrami:** Methodology; investigation; writing – review and editing; visualization. **Henna Pehkonen:** Methodology; investigation; visualization; writing – review and editing. **Alessia Centanni:** Writing – review and editing; investigation. **Ahmed B. Montaser:** Investigation; methodology; writing – review and editing; formal analysis. **Arina Värä:** Writing – review and editing; investigation. **Jelena Matović:** Investigation; writing – review and editing. **Heidi Liljenbäck:** Investigation; writing – review and editing. **Tatsiana Auchynnikava:** Investigation; writing – review and editing. **Kristiina M. Huttunen:** Methodology; supervision; funding acquisition. **Anne Roivainen:** Funding acquisition; resources; writing – review and editing. **Anu J. Airaksinen:** Funding acquisition; resources; writing – review and editing. **Filip S. Ekholm:** Conceptualization; funding acquisition; resources; writing – review and editing; methodology; supervision. **Outi Monni:** Conceptualization; funding acquisition; supervision; writing – review and editing; resources. **Jarkko Rautio:** Conceptualization; funding acquisition; resources; supervision; methodology; writing – review and editing. **Mirkka Sarparanta:** Conceptualization; investigation; funding acquisition; supervision; methodology; project administration; writing – review and editing; formal analysis.

## FUNDING INFORMATION

The authors gratefully acknowledge financial support from the Alfred Kordelin Foundation (grant no. 220147; SI), the Research Council of Finland (decision nos. 341106; FSE, 343608; AJA, 350117; AR, 355312; JR, 318422, 320102, and 346122; MS), Cancer Foundation Finland (Syöpäsäätiö), the Jane and Aatos Erkko Foundation, the Päivikki and Sakari Sohlberg Foundation, and the Ruth och and Nils‐Erik Stenbäcks Stiftelse Foundation.

## CONFLICT OF INTEREST STATEMENT

The authors declare no conflict of interest.

## ETHICS STATEMENT

All animal experiments in this study were conducted under the approved project licenses ESAVI/12132/04.10.07/2017, ESAVI/12269/2020, and ESAVI/9782/2022 granted by the National Board of Animal Experimentation in Finland and followed European Union legislation (Directive 2010/63/EU).

## Supporting information


**DATA S1.** Supporting information.

## Data Availability

The presented data is available from the corresponding author upon reasonable request.
