# Supplementary material for: Biological evaluation of a glucose‐based boron carrier as a potential agent for boron neutron capture therapy
Source: Int J Cancer. 2025 Jul 23;157(11):2374–84. doi: 10.1002/ijc.70054 (PMC12496008; doi:10.1002/ijc.70054)
Supplement: Supplementary file 1 — DATA S1. Supporting information. [file IJC-157-2374-s001.pdf]

Supporting Information

Biological Evaluation of a Glucose-Based Boron Carrier as a Potential Agent for Boron Neutron Capture Therapy

Surachet Imlimthan, Katayun Bahrami, Henna Pehkonen, Alessia Centanni, Ahmed B. Montaser, Arina Värä, Jelena Matović, Heidi Liljenbäck, Tatsiana Auchynnikava, Kristiina M. Huttunen, Anne Roivainen, Anu J. Airaksinen, Filip S. Ekholm, Outi Monni, Jarkko Rautio, Mirkka Sarparanta

Table of contents:

Supplementary Information on materials and methods

Supplementary Tables S1 – S3

Supplementary Figures S1 – S8

Supplementary References

Experimental animals

Generally, animals were housed in conventional polysulfone cages supplied with aspen bedding (Tapvei®, Harjumaa, Estonia), and enrichments (e.g., nesting material and disposable cardboard hut). Pelleted food (Teklad 2019C diet, Envigo, Huntingdon, UK) and water were provided *ad libitum*; moreover, diet gel (Solid Drink® SDDGC-56, Triple A Trading, Tiel, the Netherlands) was supplied inside the cage after tumor inoculation. Animal housing environment was maintained at 12/12 h dark/light cycle, 22±1 °C, and 55±15% relative humidity throughout the studies. The CAL 27 and UT-SCC-14 tumor models were established in female athymic nude Rj:ATHYM-*Foxn1*$^{nu/nu}$ mice, aged 12 weeks, and weighing 16–20 g (Janvier Laboratories, Le Genest-Saint-Isle, France).

Table S1: Biodistribution results of [^{18}F]FDG at 60-min post-injection

Tissue / Condition	CAL 27 tumor-bearing animals				UT-SCC-14 tumor-bearing animals			
	Fasting		Non-fasting		Fasting		Non-fasting	
	%ID/g	cal. SUV*	%ID/g	cal. SUV*	%ID/g	cal. SUV*	%ID/g	cal. SUV*
**Urine**	286.77±318.53	55.21±59.69	152.40±127.86	31.78±26.97	147.61±112.34	25.79±16.80	57.82±12.40	11.23±1.79
**Blood**	0.76±0.11	0.15±0.02	0.50±0.13	0.10±0.02	0.90±0.33	0.16±0.04	0.57±0.01	0.11±0.01
**Gallbladder**	1.67±0.42	0.33±0.08	1.26±0.26	0.26±0.06	1.65±0.44	0.31±0.18	1.31±0.52	0.26±0.12
**Liver**	0.99±0.06	0.20±0.02	0.75±0.23	0.15±0.04	0.96±0.11	0.18±0.01	1.07±0.04	0.21±0.02
**Kidney**	2.04±0.81	0.40±0.16	1.30±0.46	0.27±0.10	2.20±1.06	0.39±0.14	1.72±0.07	0.34±0.03
**Muscle**	8.14±0.47	1.60±0.04	5.69±1.40	1.17±0.23	7.54±0.64	1.39±0.08	8.27±1.63	1.61±0.23
**Tumor**	7.45±1.06	**1.47±0.23**<br>**1.02±0.34****	7.24±1.05	**1.51±0.28**<br>**0.96±0.16****	8.48±2.68	**1.53±0.27**<br>**1.02±0.25****	7.13±1.02	**1.40±0.28**<br>**0.97±0.27****
**Tongue**	9.79±4.03	1.95±0.90	11.24±0.83	2.33±0.25	10.09±0.09	1.87±0.25	7.71±2.27	1.49±0.36
**Mucous membrane**	7.14±0.60	1.41±0.19	5.03±2.01	1.03±0.36	6.01±0.33	1.12±0.22	4.78±0.63	0.94±0.18
**Brain**	8.04±0.74	1.58±0.13	5.82±0.30	1.21±0.11	9.12±4.95	1.62±0.67	6.29±0.01	1.23±0.07

**cal. SUV = calculated SUV from biodistribution data = [%ID/g]/[body weight (g)/100]*

***SUV values obtained from PET/CT image analysis by drawing the region of interest (ROI) around the tumor area*

Sample preparation for MP-AES measurement

All collected tissue samples underwent acid digestion with a 3 ml of 70% nitric acid (HNO_3), except liver (20 ml) at 80 °C for 4–5 h until the samples clarified, and no residues were visible. After the digested samples cooled down to room temperature, 0.5–1.5 ml was transferred to a new 15-ml conical tube and diluted with deionized water to constitute the concentration of HNO_3 to ≤ 20% (v/v), while maintaining a constant total volume of 5 ml in all samples (refer to the sample preparation table in supporting information **Table S2**). Subsequently, 25 μl of 1000 ppm scandium internal standard (ROMIL, Cambridge, UK) and 50 μl of 100 mg/ml cesium ionization buffer in 5% HNO_3 (Agilent Technologies, Santa Clara, CA, USA) were added to each diluted sample. Samples were passed through 0.22-μm filter before measurement. The detection wavelengths for boron and scandium are 249.772 and 361.383 nm, respectively. Boron concentration in the sample was determined using a boron standard curve (concentration range: 0–20 ppm, $R^2 > 0.9999$). The total boron concentration in tissue is expressed in parts per million (ppm).

Table S2: Sample preparation for MP-AES measurement

Tissue	Digested sample (μl)	Deionized water (μl)	Sc STD (1000 ppm, μl)	Cs buffer (μl)	Dilution factor	Total volume (μl)
**Urine**	500	4425	25	50	10	5000
**Blood**	500	4425	25	50	10	5000
**Gallbladder**	500	4425	25	50	10	5000
**Liver**	500	4425	25	50	10	5000
**Kidney**	500	4425	25	50	10	5000
**Muscle**	500	4425	25	50	10	5000
**Tumor**	1500	3425	25	50	10/3	5000
**Tongue**	1500	3425	25	50	10/3	5000

**Mucous membrane**	1500	3425	25	50	10/3	5000
**Brain**	1500	3425	25	50	10/3	5000

Immunohistochemistry

UT-SCC-14 and CAL 27 tumors were excised and immediately submerged into liquid nitrogen for snap freezing or fixed with 4% formaldehyde solution (Sigma-Aldrich, St. Louis, MO, USA) before further analysis. Before sectioning, tumors were processed through a series of rinses and ethanol incubations as follows: 1) rinsed with PBS, 2) incubated in 50% ethanol, 3) rinsed again with PBS, and 4) incubated in 70% ethanol. Tumors were embedded in paraffin blocks and cut into 4-μm sections with HM 355S automatic microtome (Thermo Scientific, Waltham, MA, USA). VECTASTAIN® Elite ABC-HRP-kit was used according to the manufacturer's protocol for IHC staining (Vector Laboratories, Newark, CA, USA). Paraffin sections were deparaffinized using xylenes, rehydrated through a graded alcohol series, and then washed with tap water. Slides were pre-treated with Tris-EDTA solution (pH 9). Blocking was carried out by rinsing the slides with 3% hydrogen peroxide, washing with PBS, and incubating with 20% goat serum. Rabbit polyclonal GLUT1 (21829-1-AP, Proteintech, Rosemont, IL, USA) was used as a primary antibody. Tumor sections were incubated with the primary antibody solution prepared in 1% BSA in PBS (1:1000) for 1 h. Rabbit IgG isotype control (ab37415, Abcam, Cambridge, UK) solution was prepared in 1% BSA in PBS (1:2000) and used as a negative control. Tumor sections were then incubated with goat anti-rabbit IgG secondary antibody (H+L), biotinylated (BA-1000, Vector Laboratories, Newark, CA, USA) diluted in 1% BSA in PBS (1:200) for 30 min. After rinsing with PBS, sections were incubated with VECTASTAIN® Elite ABC reagent for 30 min to allow ABC complexation, followed by washing with PBS. The slides were imaged using the Pannoramic 250 FLASH III digital slide

scanner (3DHISTECH kft., Budapest, Hungary). Images were processed using SlideViewer 2.7, Adobe Photoshop, and Illustrator CC (Adobe Inc., San Jose, CA, USA).

Cell culture materials and reagents

Cell culture flasks and well plates were purchased from Corning Inc. (Corning, NY, USA). Essential cell culture media and additives, including Dulbecco's phosphate-buffered saline (10×DPBS), Dulbecco's Modified Eagle's Medium (DMEM) for CAL 27, UT-SCC-28, and UT-SCC-42B cells, Dulbecco's Modified Eagle's Medium:Nutrient Mixture F-12 (DMEM:F-12) for UT-SCC-14 cell, heat-inactivated fetal bovine serum (FBS), Penicillin-Streptomycin (10,000 U/ml), GlutaMAX (100×), sodium pyruvate (100 mM), TrypLE™ Express (1×), MEM nonessential amino acid (100×), and individually-wrapped sterile vacuum filter/storage bottle (250/500 ml, 0.22 μm PES membrane) were procured from Life Technologies Gibco (Carlsbad, CA, USA) and Lonza Group AG (Basel, Switzerland). Keratinocyte serum-free medium (1×SFM), human recombinant epidermal growth factor (rEGF), and bovine pituitary extract (BPE) for CCD 1106 cell culture were acquired from Thermo Fisher Scientific (Waltham, MA, USA). The EVE™ automated cell counter and cell counting kit were obtained from NanoEnTek Inc. (Guro-gu, Seoul, Korea).

General cell culture procedure

All cell culture procedures were carried out in a biosafety cabinet under aseptic condition. All working reagents and solutions were pathogen-free and sterile filtered. Cell culture media for all HNSCC cell lines were typically supplemented with 10% FBS, 1×GlutaMAX, 1 mM sodium pyruvate, and 1% Penicillin-Streptomycin, and aseptically filtered through sterile 0.22-μm filter before use. CCD 1106 cell line was cultured in keratinocyte 1×SFM, supplemented

with rEGF, BPE, and 1% Penicillin-Streptomycin at the time of use. Cell culture was maintained in a humidified cell incubator at 37 °C and 5% CO_2 atmosphere.

General cell preparation protocol

Cells in the culturing flask were washed twice with 1×DPBS before being treated with 1×TrypLE™ Express to facilitate cell detachment in an incubator. The cell suspension was neutralized with fresh medium before centrifugation (900 rpm, 5 min) to collect the cell pellet. The cell pellet was redispersed with fresh medium, and the total cell number was determined using the cell counting kit as directed by the manufacturer protocol. Cells were then prepared for subsequent *in vitro* and *in vivo* assays. If the medium needed to be changed (for example to 1×DPBS for tumor implantation), centrifugation was used to collect the cell pellet.

Western blotting

Western blotting was conducted as described earlier.[1] The cells were lysed using RIPA lysis buffer (Sigma-Aldrich, St. Louis, MO, USA) supplemented with phosphatase and protease inhibitors (Roche, Basel, Switzerland). Lysate protein concentration was measured with the BCA protein assay kit according to manufacturer's instructions (Thermo Fisher Scientific, Waltham, MA, USA). Proteins were transferred to polyvinylidene fluoride (PVDF) membrane using Trans-Blot Turbo equipment (Bio-Rad, Hercules, CA, USA). Blocking of the membrane was done in 5% BSA in Tris-buffered saline and Tween® 20 (TBST) solution. GLUT1 sheep polyclonal antibody (ab54263, Abcam, Cambridge, UK) was diluted in 1% BSA in TBST (1:1000), and the membrane was incubated overnight. Anti-Vinculin mouse monoclonal antibody (V9264, Merck, Rahway, NJ, USA) was used as a loading control, diluted in 1% BSA in TBST solution (1:1000). Anti-sheep IgG secondary antibody (81-8620, Invitrogen, Waltham, MA, USA) and anti-mouse IgG secondary antibody (62-6520, Invitrogen, Waltham, MA, USA)

were diluted in 1% BSA in TBST solution (1:10000). Membrane was washed with TBST solution. Detection reagents for chemiluminescence were obtained from Merck-Millipore (Burlington, MA, USA). CHEMIDOC (Bio-Rad, Hercules, CA, USA) and Adobe Photoshop and Illustrator CC (Adobe Inc., San Jose, CA, USA) were used for visualization of the results.

Proteomic analysis

UT-SCC-14, UT-SCC-28, and UT-SCC-42B cells were cultured in 15-cm tissue culture dishes. Cells were washed three times with ice-cold 1×PBS (pH 7.4) and scraped off using a cell scraper. Plasma membranes were extracted from the cell lysates using the Membrane Protein Extraction Kit (BioVision Inc., Milpitas, CA, USA) following the manufacturer's instructions, and stored at -80 °C until further analysis. Plasma membrane fractions were solubilized in 2% SDS solution and incubated at 95 °C for 5 min. Protein content from each fraction was quantified using the Pierce™ BCA Protein Assay Kit (Thermo Fisher Scientific, Waltham, MA, USA). A total of 50 µg of protein from each fraction was then denatured, reduced, and alkylated according to the filter-assisted sample preparation (FASP) protocol as previously described.[2,3] Briefly, the buffer was exchanged for 8 M urea, and proteins were reduced with 0.1 M dithiothreitol (Merck, Darmstadt, Germany) for 1 hour at RT while mixing at 500 rpm, followed by alkylation with 0.05 M iodoacetamide (Merck, Darmstadt, Germany) for 30 min in the dark using a Microcon centrifugal filter (MWCO 30 kDa, Merck Millipore, Darmstadt, Germany). The samples were then washed twice with 8 M urea by centrifugation at 14,000*g* for 15 min at RT. Next, 48 µl of 0.05 M ammonium bicarbonate in ultrapure water spiked with 50 fmol of isotopically labeled heavy peptide, 1:100 (w/w) endoproteinase LysC (Promega, WI, USA), and 0.05% ProteaseMax (Promega, WI, USA) was added to the filters and incubated at 30 °C for 3 h. The proteins were then digested with TPCK-treated trypsin (1:100 w/w; Promega Biotech AB, Nacka, Sweden) at 37 °C for 18 h. The resulting peptides were recovered

by centrifugation at 14,000*g* for 10 min, followed by two additional elutions with 50 µl of 50% acetonitrile in ammonium bicarbonate buffer. The solvent was evaporated using a SpeedVac concentrator (Thermo Fisher Scientific, MA, USA) at RT, and samples were resuspended in 50 µl of 2% acetonitrile mixed 5% formic acid solution in ultrapure water. The digested peptides from each sample were analyzed using an ultra-performance liquid chromatography (UPLC) coupled with a triple quadrupole mass spectrometer with a heated electrospray ionization source in positive mode (UPLC 1290 and MSD 6495, Agilent Technologies, Santa Clara, CA, USA) as previously described.[3] A total of 10 µg of digested peptides (protein equivalent) and 25 fmol of heavy peptide were injected into the LC-MS/MS and separated using an AdvanceBio Peptide Map column (2.1×250 mm, 2.7 µm, Agilent Technologies). Peptide separation was achieved using a gradient of 0.1% formic acid in water (A) and acetonitrile (B) at a constant flow rate of 0.3 ml/min with the following gradient: 2–7% B for 2 min, 7–30% B for 48 min, 30–45% B for 3 min, 45–80% B for 2.5 min, followed by re-equilibration for 4.5 min. Protein quantification was based on the ratio of light to heavy standard peptides as previously reported in **Table S3**.[4,5] Data were acquired using Agilent MassHunter software (version B.03.01) and processed with Skyline software (version 20.1).

Table S3: MRM/SRM transitions for absolute quantitative proteomics[4,5]

**Protein**	**Gene**	**Peptide**	**Type**	**Retention time (min)**	**Precursor ion**	**Product ion**		
					**Q1**	**Q3-1**	**Q3-2**	**Q3-3**
GLUT1	*SLC2A1*	TFDEIASGFR	St	30.4	571.78	894.43	537.28	650.36
		TFDEI**A***SGFR	SIS		573.78	898.44	541.29	654.37

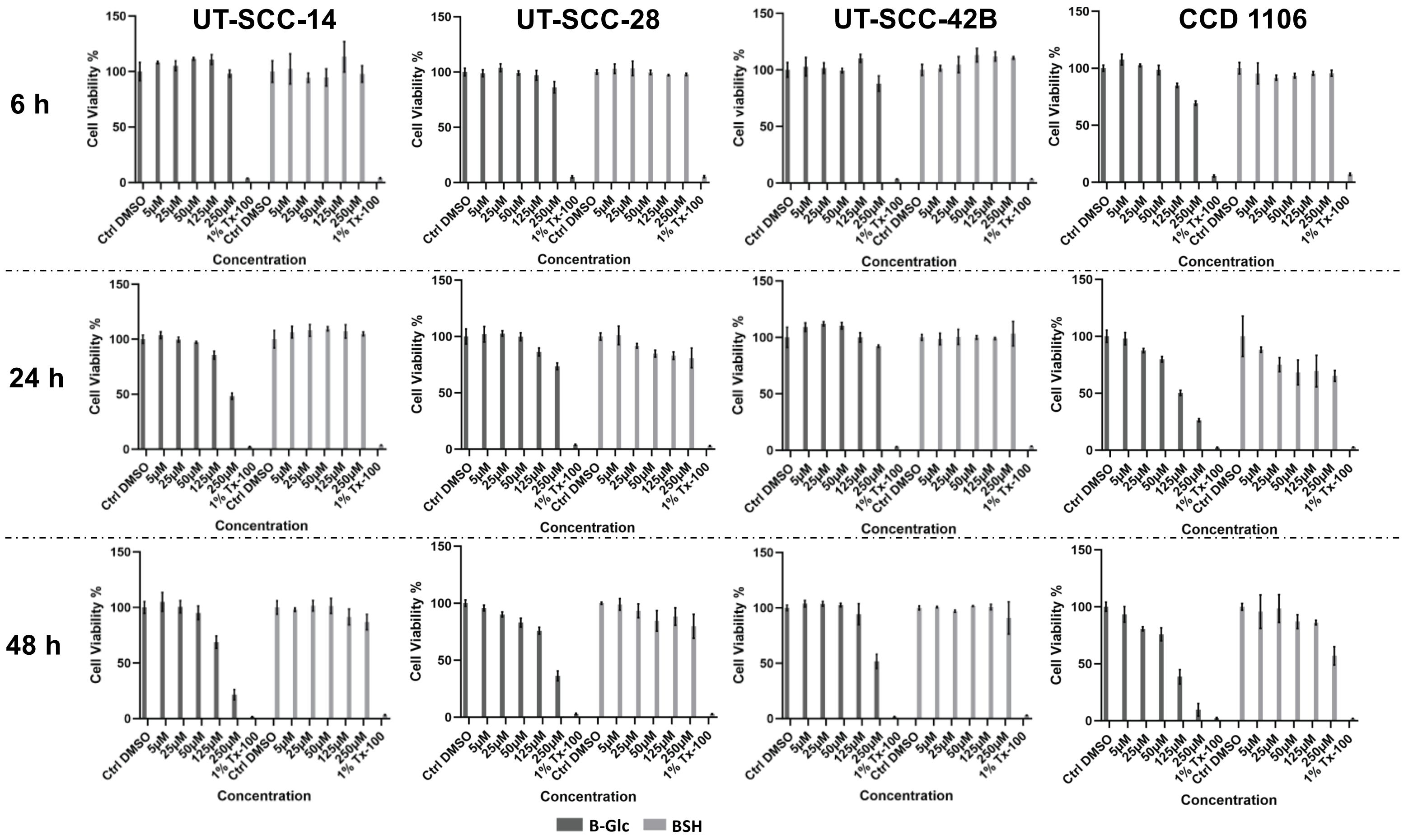

Figure S1: *In vitro* cytotoxicity of the B-Glc and BSH (a clinically relevant boron-delivery control) evaluated at concentrations of 5, 25, 50, 125, and 250 μM over 6 h (top panel), 24 h (middle panel), and 48 h (bottom panel) in HNSCC UT-SCC-14, UT-SCC-28, UT-SCC-42B, and keratinocyte CCD 1106 (healthy cell control) cell lines. Negative and positive controls for cell viability were 0.1% (v/v) DMSO in complete cell culture medium and 1% (v/v) Triton X-100 solution, respectively. Data are presented as mean ± s.d. (n = 3).

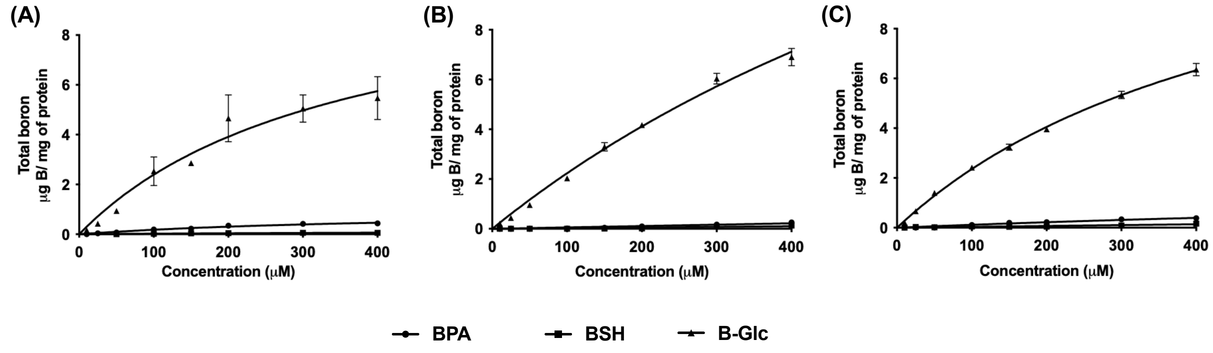

Figure S2: Cell uptake after 30 min of incubation with the B-Glc (▲), BPA (●), and BSH (■) in (**A**) UT-SCC-14, (**B**) UT-SCC-28, and (**C**) UT-SCC-42B cells at a concentration of 10–400 µM (n = 3).

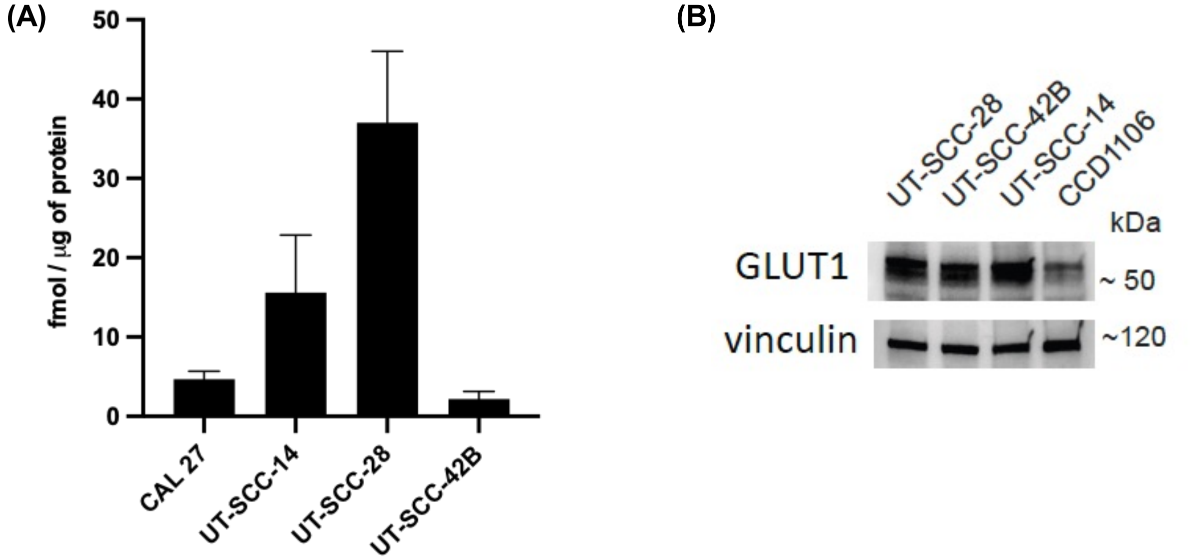

Figure S3: (**A**) Proteomic analysis of GLUT1 expression in HNSCC CAL 27, UT-SCC-28, UT-SCC-14, and UT-SCC-42B cell lines. GLUT1 expression data for CAL 27 cell line is taken from our previous report.[6] Protein levels were analyzed from plasma membrane fractions and are presented as average ± s.d. (n = 3, except for UT-SCC-42B, where n = 2). (**B**) Western blot analysis conducted to assess GLUT1 expression in HNSCC UT-SCC-28, UT-SCC-14, UT-SCC-42B, and healthy control keratinocyte CCD 1106 cell lines. GLUT1 and vinculin (housekeeping control) proteins were detected at molecular weights of ~50 kDa and ~120 kDa, respectively.

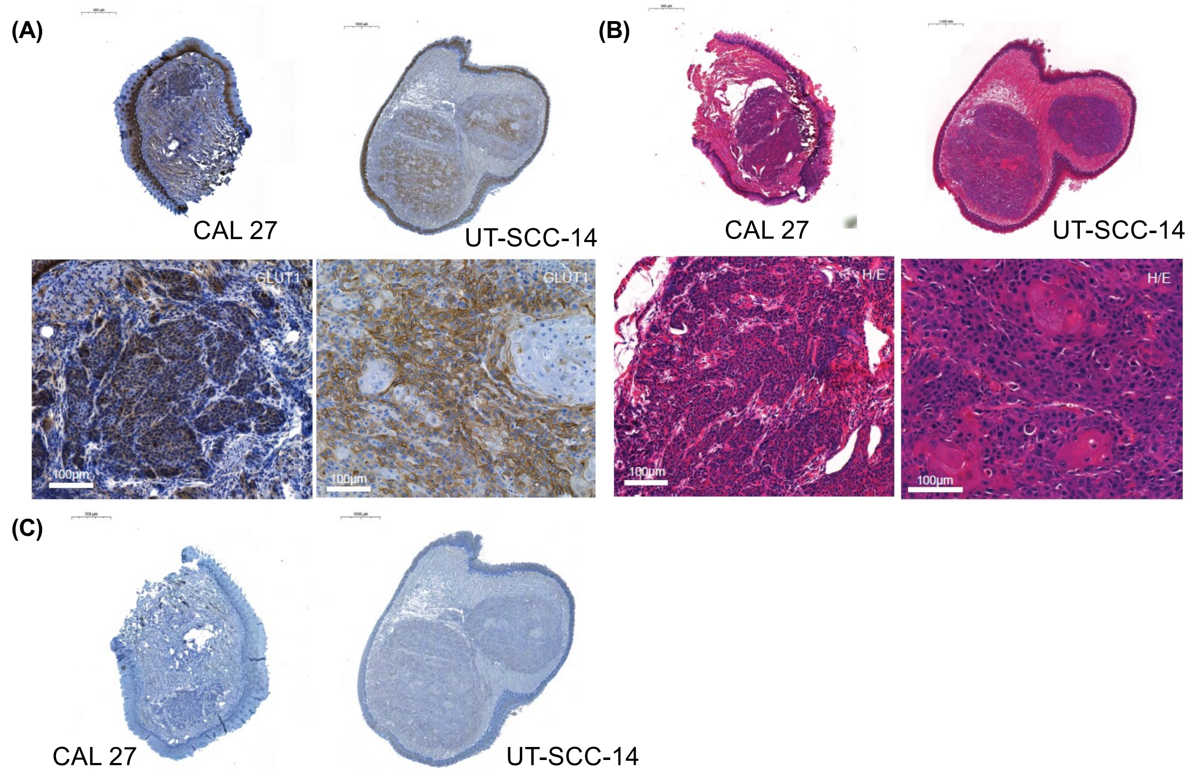

Figure S4: (**A**) Immunohistochemical images illustrating GLUT1 expression in untreated CAL 27 (left) and UT-SCC-14 (right) tumors. (**B**) Histological images showing H&E staining sections of CAL 27 (left) and UT-SCC-14 (right) tumors. (**C**) Immunohistochemical images of tumor sections from CAL 27 (left) and UT-SCC-14 (right) stained with rabbit IgG isotype served as a negative control. All slides were scanned at 20× magnification. The scale bar for the entire tumor section was 500 µm for CAL 27 and 1000 µm for UT-SCC-14 tumor sections. Images in the lower panels of **A** and **B** were magnified, with a scale bar of 100 µm.

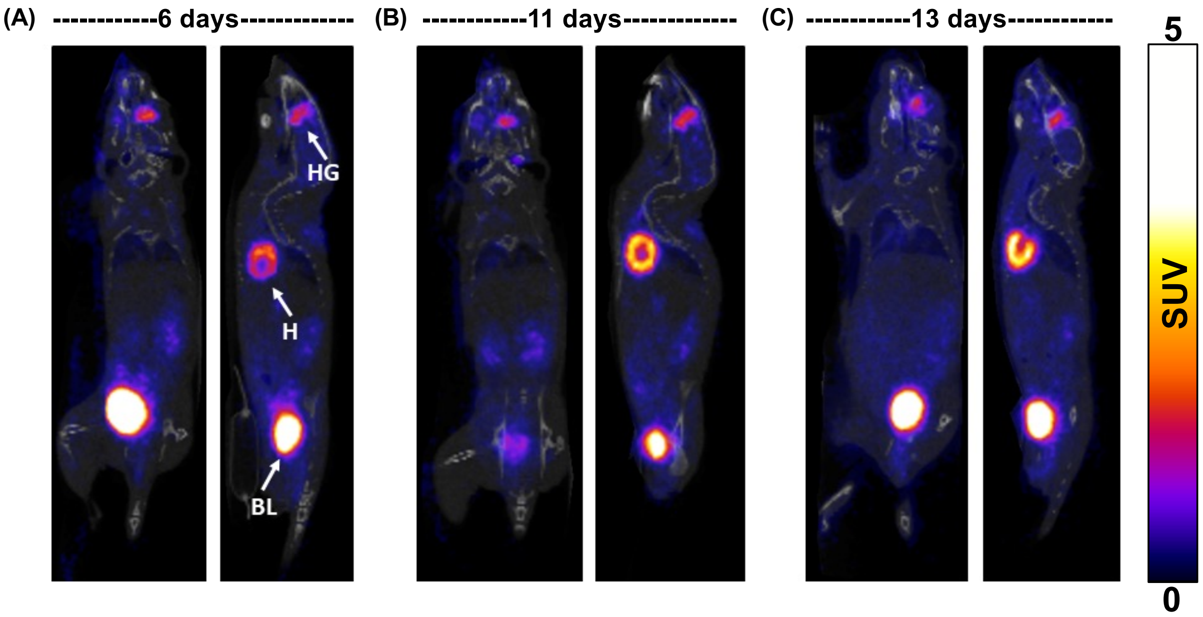

Figure S5: Representative 90-min dynamic PET/CT images in coronal (left) and sagittal (right) planes after [^{18}F]FDG administration in UT-SCC-14 tumor-bearing mice following 3-hour fasting and administration of the tracer under isoflurane anesthesia at (**A**) 6 days, (**B**) 11 days, and (**C**) 13 days after tumor implantation. HG denotes harderian gland, H for heart, and BL for bladder.

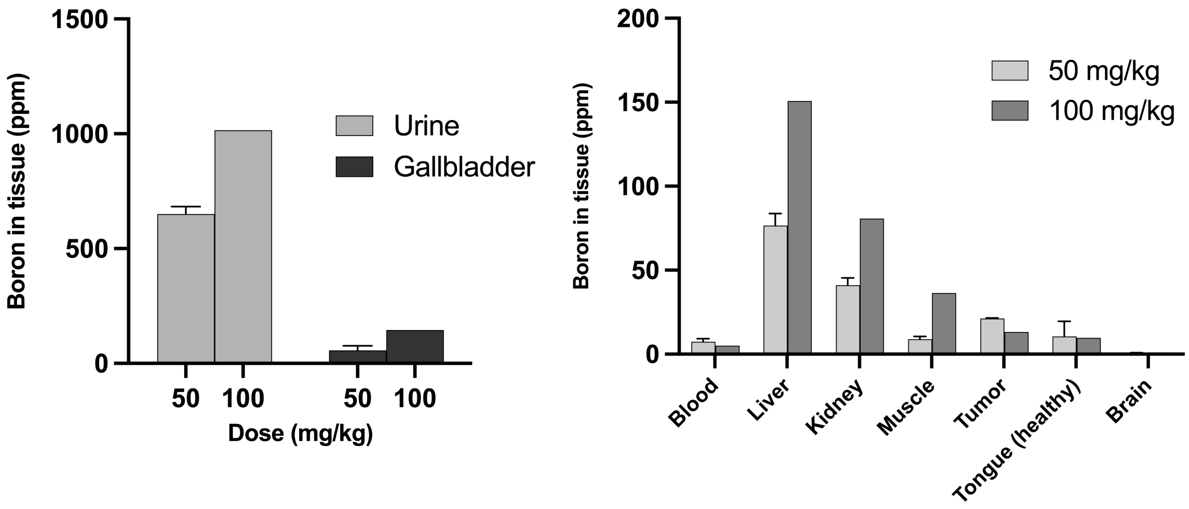

Figure S6: Biodistribution profiles after the administration of the B-Glc in non-fasting CAL 27 tumor-bearing mice at 60 min. The values represent the mean ± s.d. (n=1–2) in parts per million (ppm).

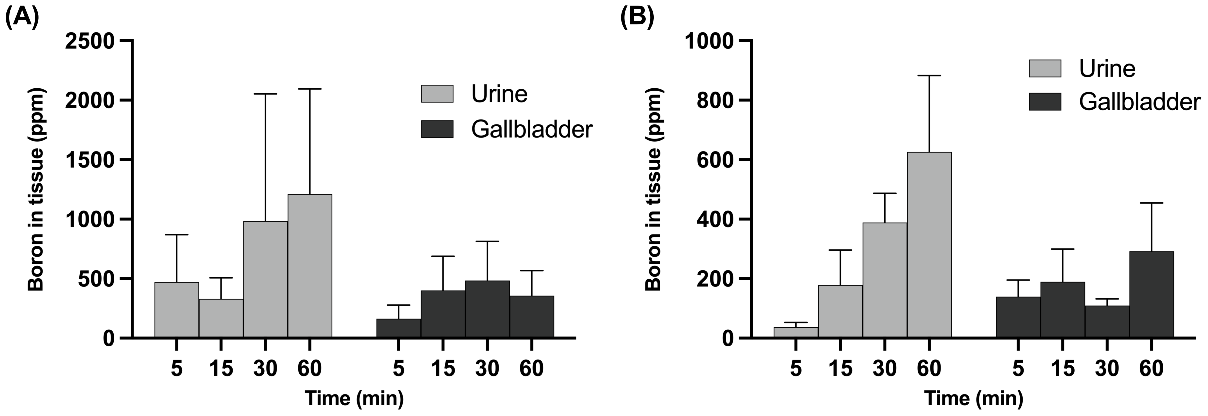

Figure S7: Urine and gallbladder excretions of B-Glc at 75 mg/kg in UT-SCC-14 tumor-bearing animals in (**A**) non-fasting and (**B**) fasting groups at 5, 15, 30, and 60 min. The values represent the mean ± s.d. (n=3–4) in parts per million (ppm).

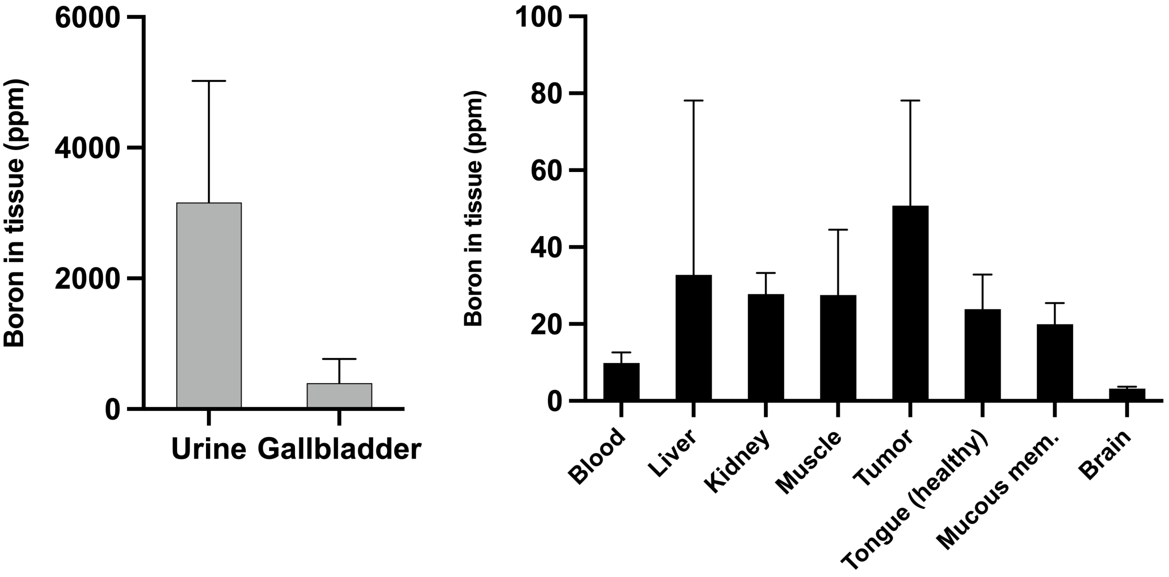

Figure S8: Biodistribution of BPA-F at 400 mg/kg in fasting UT-SCC-14 tumor-bearing animals at 60 min. The values represent the mean ± s.d. (n=4) in parts per million (ppm).

References

[1] Pehkonen, H.; Filippou, A.; Väänänen, J.; Lindfors, I.; Vänttinen, M.; Ianevski, P.; Mäkelä, A.; Munne, P.; Klefström, J.; Toppila-Salmi, S.; Grènman, R.; Hagström, J.; Mäkitie, A. A.; Karhemo, P.-R.; Monni, O. Liprin-α1 Contributes to Oncogenic MAPK Signaling by Counteracting ERK Activity. *Mol Oncol* **2024**. *18*(3), 662–676. https://doi.org/10.1002/1878-0261.13593

[2] Wiśniewski, J. R. Filter Aided Sample Preparation – A Tutorial. *Anal Chim Acta* **2019**. *1090*, 23–30. https://doi.org/j.aca.2019.08.032

[3] Ramsay, E.; Montaser, A. B.; Niitsu, K.; Urtti, A.; Auriola, S.; Huttunen, K. M.; Uchida, Y.; Kidron, H.; Terasaki, T. Transporter Protein Expression of Corneal Epithelium in Rabbit and

Porcine: Evaluation of Models for Ocular Drug Transport Study. *Mol Pharm* **2024**. *21*(7), 3204–3217. https://doi.org/10.1021/acs.molpharmaceut.3c01210
[4] Uchida, Y.; Yagi, Y.; Takao, M.; Tano, M.; Umetsu, M.; Hirano, S.; Usui, T.; Tachikawa, M.; Terasaki, T. Comparison of Absolute Protein Abundances of Transporters and Receptors among Blood–Brain Barriers at Different Cerebral Regions and the Blood–Spinal Cord Barrier in Humans and Rats. *Mol Pharm* **2020**. *17*(6), 2006–2020. https://doi.org/10.1021/acs.molpharmaceut.0c00178
[5] Uchida, Y.; Zhang, Z.; Tachikawa, M.; Terasaki, T. Quantitative Targeted Absolute Proteomics of Rat Blood–Cerebrospinal Fluid Barrier Transporters: Comparison with a Human Specimen. *J Neurochem* **2015**. *134*, 1104–1115. https://doi.org/10.1111/jnc.13147
[6] Matović, J.; Järvinen, J.; Sokka, I. K.; Imlimthan, S.; Raitanen, J.-E.; Montaser, A.; Maaheimo, H.; Huttunen, K. M.; Peräniemi, S.; Airaksinen, A. J.; Sarparanta, M.; Johansson, M. P.; Rautio, J.; Ekholm, F. S. Exploring the Biochemical Foundations of a Successful GLUT1-Targeting Strategy to BNCT: Chemical Synthesis and *In Vitro* Evaluation of the Entire Positional Isomer Library of *ortho*-Carboranylmethyl-Bearing Glucoconjugates. *Mol Pharm* **2020**. *18*(1), 285–304. https://doi.org/10.1021/acs.molpharmaceut.0c00917
